# The Predictive Capacity of Air Travel Patterns during the Global Spread of the COVID-19 Pandemic: Risk, Uncertainty and Randomness

**DOI:** 10.3390/ijerph17103356

**Published:** 2020-05-12

**Authors:** Panayotis Christidis, Aris Christodoulou

**Affiliations:** Directorate C: Energy and Transport, Joint Research Centre, European Commission, c/Inca Garcilaso 3, ES-41092 Sevilla, Spain; Aris.Christodoulou@ec.europa.eu

**Keywords:** air transport, aviation, COVID-19, coronavirus, epidemic model, risk assessment, spatial analysis

## Abstract

Air travel has a decisive role in the spread of infectious diseases at the global level. We present a methodology applied during the early stages of the COVID-19 pandemic that uses detailed aviation data at the final destination level in order to measure the risk of the disease spreading outside China. The approach proved to be successful in terms of identifying countries with a high risk of infected travellers and as a tool to monitor the evolution of the pandemic in different countries. The high number of undetected or asymptomatic cases of COVID-19, however, limits the capacity of the approach to model the full dynamics. As a result, the risk for countries with a low number of passengers from Hubei province appeared as low. Globalization and international aviation connectivity allow travel times that are much shorter than the incubation period of infectious diseases, a fact that raises the question of how to react in a potential new pandemic.

## 1. Introduction

The recent pandemic caused by the novel coronavirus COVID-19 has once again brought epidemiological modeling into the limelight. Especially during the early stages of the virus propagation—when data were limited and highly uncertain—model projections were a major input for understanding the magnitude and potential risks of the outbreak. Apart from predicting the dynamics of transmission within a specific population, particular emphasis was put on quantifying the risk of the disease spreading beyond the area of its original focus in Wuhan, China.

The contribution of transport—and aviation in particular—has been shown to be significant in the spread of airborne diseases such as SARS, MERS [1,2] and the influenza A H1N1 [3], as well as other infectious such as Ebola [4] or even vector-borne diseases such as Dengue [5] or Malaria [6].

Spatio-temporal dynamics also have a major role. Social interactions and personal contact patterns at either the origin of the disease or areas connected to it affect the speed and range of prevalence across different geographic zones [7]. Budd et al. [8] made a historical review of journey times and the incubation period of selected infectious diseases. Travel times have decreased drastically and even intercontinental trips have a duration that is a fraction of the incubation period of several diseases. Coming to the case of the COVID-19 outbreak, no intercontinental flights from Wuhan were possible before 2012, but in 2020 direct flights were available to London, Paris, Rome, Moscow, New York City, San Francisco, Istanbul, Dubai, Sydney, and numerous other international destinations. As an example, a direct flight from Wuhan to Rome, Italy takes 12 h 15 m and is available three times weekly.

Several studies have utilized air travel data for the estimation of disease importation risks. The Vector-Borne Disease Airline Importation Risk (VBD-AIR) tool allows the combination of disease information and air travel networks to produce a user-defined global map of risk distribution [9]. A generic tool that allows the estimation of the median early disease arrival time from around the world using air transport schedules was proposed by [10]. Air travel patterns were also employed by Bajardi et al. [11] in order to theoretically model the potential impacts of travel restrictions on the spread of an epidemic. Nevertheless, while travel and trade have been shown as relevant for the international spread of the 2009 A/H1N1 influenza, the slow deployment of control measures in countries with lower healthcare capacities led to spatial imbalances [12]. Air transport data have been used indirectly to measure the effective distance and the relative arrival time of a disease outbreak, with promising results when tested on historical data [13]. Based on the same premises, producing near real-time or now-casting predictions on the international spread of COVID-19 appeared to be based on scientifically sound principles [14].

However, most expectations about the ability to predict the global spread of COVID-19 proved misleading or of limited use.

This includes our own work, which we present here in order to discuss the reasons why most projections missed important aspects of the disease propagation dynamics. In our opinion, and as a part of the scientific process, it is important to publish and discuss even the negative results of research activities. The lessons learned from unsuccessful applications can be valuable for future work, either by improving current practices or by re-examining risk assessment from a new perspective. Especially in the case of predictive modeling, the wide body of literature that presents successful ex-post examples of possible value may give the false impression that the spatial aspects of disease propagation are more easily predictable than what is actually possible in reality.

The question this paper is trying to answer is whether air passenger traffic alone can provide early information on the potential distribution of contagious diseases at the global level. Based on the experience from the SARS, MERS and Ebola epidemics—which suggests that travel patterns are a good predictor of spatial evolution of an epidemic—we mapped the expected distribution of COVID-19 at the global level using now-casted origin-destination estimates of aviation passengers and near real-time data on the spatial distribution of diagnosed infections. The number of reported cases for each possible destination outside the initial outburst area was compared to the estimated traffic intensity between Wuhan and the specific destination. The resulting ratio was used as an indicator of the expected rate of infection of aviation passengers and as a means of comparison of the evolution of the pandemic worldwide. The approach gave promising results during the initial phases of the pandemic, but underestimated the risks associated with local infections at several distant destinations and the development of numerous secondary foci of the disease.

The structure of the paper is as follows: Section 2 describes the main elements of the modeling approach followed. Section 3 discusses the results and Section 4 analyzes the factors limiting the predictive capacity. Finally, Section 5 summarizes the conclusions of this work.

## 2. Modeling the Risk of Coronavirus Spread Using Aviation Data

The information on the characteristics of the virus during the initial phases of the pandemic, in January and February 2020, was very limited and arguably imprecise. Given that the vast majority of diagnosed cases at that stage were concentrated in China (99% on 3 February), estimating the risk for other countries in real time was—at least in retrospect—practically impossible. The distribution of the cases within China appeared to follow a distribution similar to a gravity model, an observation that suggested that travel patterns—which also present gravity model patterns of spatial distribution—may be used as a proxy for the expected appearance of COVID-19 outside China.

The main hypothesis we made was that each passenger travelling from Hubei province after the initial outbreak would have the same risk of being infected at a specific point in time, regardless of the destination. Since the basic epidemiological analysis shows that the risk increases over time, a similar increase would be expected for all destinations. In principle, even if the risk level itself is unknown, the ratio of diagnosed cases to number of passengers should be comparable across destinations at any point in time.

### 2.1. Analyzing Air Transport Patterns

The first step of the approach was the estimation of the number of passengers leaving Hubei province by plane and reaching a specific final destination, including possible connections. Wuhan is an aviation hub in central China [15]. Wuhan and Hubei province are well connected with neighboring provinces by rail and coach [16]. We used monthly data from the SABRE database [17,18] to track the number of passengers to all possible destinations using historical data from 2016 to November 2019, the last month for which data were available at the time this paper was written. Almost 1 million passengers flew out of Wuhan every month. While China represents 89.9% of the final destinations, indirect connections linked Hubei province to 23,532 distinct airports across the world. Air transport activity from Wuhan, as across the aviation sector in general, is quite volatile with important fluctuations over time and a high seasonal variation [19]. In addition, January and early February traffic is greatly affected by the Chinese New Year’s holiday, both in terms of reduced economic activity and increased tourism.

**Figure 1:**
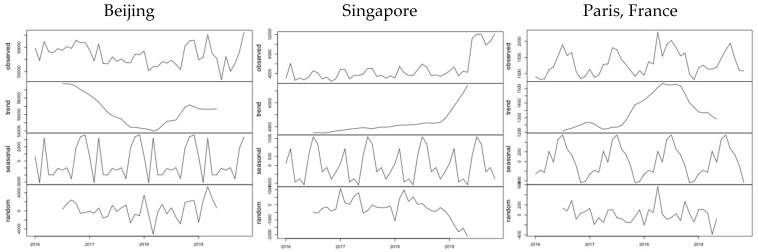
Example of decomposition of time series for three airports.

In order to create a more realistic picture of the number of passengers who left Wuhan during January 2020, we developed a now-casting model to estimate the expected numbers of passengers based on the trends revealed by data until November 2019. We applied an ARIMA (Autoregressive Integrated Moving Average) model for each of the 23,532 possible destinations. The model was estimated using the forecast package in R [20]. The decomposition of the monthly time series from January 2016 to November 2019 allows the estimation of the contribution to the observed number of passengers for each destination of the underlying trend in activity, the seasonal variation and the random effects. The examples in Figure 1 correspond to three characteristic destinations: Beijing (the top destination from Wuhan), Singapore (the top destination outside China) and Paris (the top destination outside Asia). It is evident that the three examples follow different patterns as regards both the trend in demand and the monthly variation, due to the different factors that affect international air transport demand [21].

The model we applied uses a rolling window of 36 months of historical data and a prediction step of 2 months ahead. The results were validated by comparing the model estimates with the ground truth for the last 11 months for which data were available (January 2019 to November 2019). The accuracy of the model at the individual destination level can be considered high, with an R^2^ of 0.799, MAE of 159.04 and RMSE of 906.54. A number of graphs that show how the estimates match data at the country or continent level are provided in the Appendix A.

**Figure 2:**
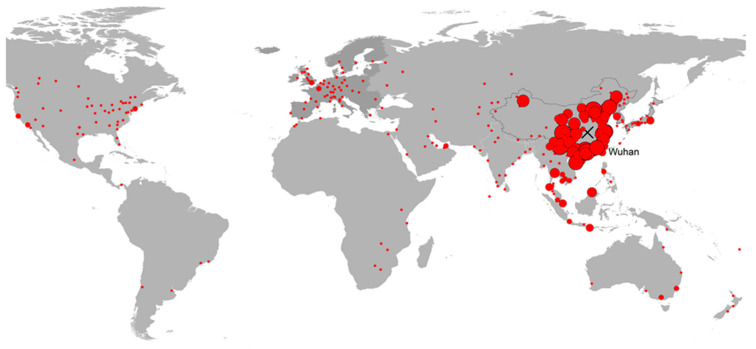
Estimated worldwide distribution of air transport passengers from Wuhan, January 2020.

Figure 2 and Table 1 summarize the results of the model at the aggregate level. For illustration purposes, China is split into three different groups based on distance from Wuhan. The distance bands (below 700 km, 700 to 1000 km and over 1000 km) correspond to the ranges where aviation has a low, moderate and high modal share [22]. The share of Chinese destinations in January 2019 was higher than their average share during the 2016–2019 period (90.5% and 89.9%, respectively), but according to the model estimates, in January 2020 it was expected to decrease to 89.4%. This would be the result of switching between various destinations, especially the increase in the number of passengers to Southeast and Far East Asia. The estimates, based on the underlying trends visible in the data available until November 2019, suggest that the share of the trips with a final destination outside Asia would remain low in January 2020, at around 1.8% of the total.

### 2.2. Combining Disease Data with Air Transport Activity

The second building block of the approach is the comparison with the numbers of diagnosed cases of COVID-19 at each potential destination. This indicator, as opposed to the number of deaths, hospitalized or recovered patients, was at the moment the only one with presumably reliable enough data in real time to derive early warning indicators on the international spread of the disease. The data we used were available, on a daily basis, through the Johns Hopkins Resource Center [23] and are summarized in Table 2, Table 3, Table 4 and Table 5.

### 2.3. A Brief Review of Epidemiological Models

The dynamics of the spread of a contagious disease are usually analyzed using either a compartmental or a phenomenological model. In the case of compartmental models, the population is divided into various groups depending on each individual’s state of infection. Such models include the SIR model (Susceptible, Infected, Recovered) and its variations, and consist of a set of differential equations that describe the change over time of the number of individuals in each compartment. In the case of the basic SIR model, the rate of change of the number of infections is expected to be:

dI/dt = β I S/N − γ I(1)

where N is the total population, I is the number of infected individuals, S is the population still susceptible to infection (total minus infected and recovered), while β and γ are parameters directly linked to the basic reproductive number of the disease R0 = β/γ.

Phenomenological models, on the other hand, attempt to simulate the disease spread by fitting data and assumptions into standard curves. A frequently used approach is the logistic equation:

dI/dt = r I_t−1_ (1 − I_t−1/_*K*). (2)

Such models rely on two parameters: the intrinsic infection rate, *r*, and the final epidemic size, K [24].

A more recent approach, the Richards curves [25], improves the matching with actual epidemiological curves, especially the early slower growth, but requires the estimation of two additional parameters:

dI/dt = r I^p^_t−1_ (1 − I_t−1_)/K^α^) (3)

where p indicates the similarity to the exponential growth curve (ranging from 0 for constant to 1 for exponential), and α is a parameter that shifts the timing of the peak.

During the early stages of an epidemic, when the population dynamics in terms of deaths and recoveries are not yet in play, the compartmental and the phenomenological approaches are in practice equivalent and the cumulative number of infections over time can be approximated by:

(4) $$\underset{t\to \infty }{\lim}\;\;I\left(t\right)=\left(1-\frac{\gamma }{\beta }\right)\;N^{}$$

Estimating the value of R_0_ (and thus the values of *β* and *γ*), or even the population *N* that should be used as a reference, is extremely challenging—if not outright futile—at the early stages of the spread of a new disease. The uncertainty and questionable accuracy of the scarce data available in the beginning of an outburst would lead to epidemiological models that are practically useless for any prediction.

### 2.4. Modelling the Spread of the Disease Based of Air Trip Patterns

Nevertheless, monitoring and comparing the spread of the disease in different locations can still provide value to public health authorities. In the approach we present here, we calculated the ratio of infected individuals to the estimated number of travellers from Hubei province to each destination. Assuming that all travellers leaving Wuhan have the same probability of being infected regardless of their destination, the cumulative number of infected individuals at time *t* reaching destination j would be expected to be:

I(*t*)_j_ = n_j_(*t*) *q* (5)

where n_j_(*t*) is the total number of passengers from the origin of the infection (Hubei province in this case) to each destination j, and q is the apparent ratio of infected passengers to total passengers:

(6) $$q=\frac{I{\left(t\right)}_{0\;}}{\sum_{j}n_{0}\left(t\right)}=\frac{I{\left(t\right)}_{0}}{v_{0}N_{0}}$$

where *I*(*t*)_0_ is the cumulative number of infected at the origin, sum(*n*_0_(*t*)) is the total number of passengers from the origin, *N*_0_ is the population at the origin and *v*_0_ is the ratio of travellers to population at the origin.

While neither formulation of Equation (6) can be directly estimated, it is sufficient to indicate that *q* is the same regardless of the destination. We should therefore expect, transforming Equation (5), that the ratio:

Q = I(t)_j_/n_j_(t) (7)

should be constant for all destinations and across time.

## 3. Discussion of the Results

We estimated q, the ratio of infected passengers to total passengers, at the province and territory level within China, and at the country level for the rest of the world. As described in Section 2, we used the estimated number of aviation passengers for January 2020 and transformed them to an equivalent daily volume. The q indicator was calculated for four different points in time at weekly intervals: on the 3rd, 10th, 17th and 24th of February 2020, corresponding to t values of 34, 41, 48 and 54 days, respectively (assuming t = 0 as 31/12/2019, the date of the first reported case in the Johns Hopkins database). The number of aviation passengers to the provinces within less than 700 km from Wuhan is virtually null (Hubei province itself, as well as Anhui, Hebei, Henan, Hunan and Jiangxi), therefore the approach is not applicable in those cases.

**Figure 3:**
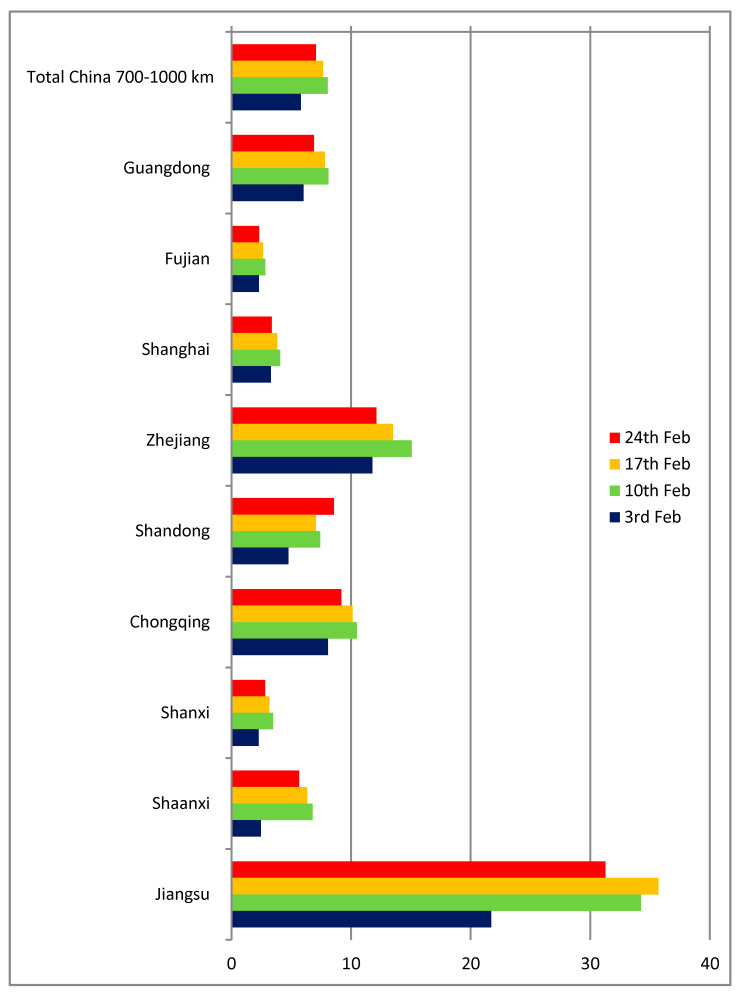
Estimated ratio of infected passengers (cases per 1000 passengers), China (700–1000 km from Wuhan).

Figure 3 shows the results for Chinese provinces between 700 and 1000 km from Wuhan. The q indicator ranges from 4 to 10 infected cases per one thousand passengers in most cases, except Zhejiang and especially Jiangsu (of which Nanjing, a major city, is only 3 h from Wuhan by train). Apart from the obvious possible differences in diagnosing and reporting, the main reason for discrepancies in the case of those provinces close to Wuhan and Hubei is their high share of train, bus and car travel to and from Wuhan.

**Figure 4:**
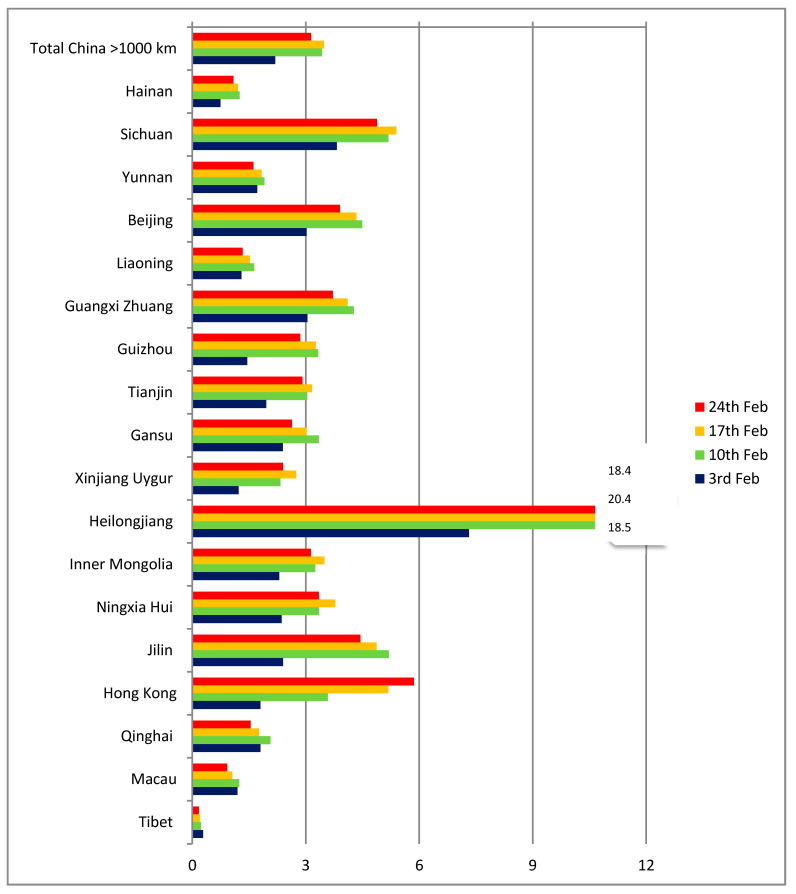
Estimated ratio of infected passengers, (cases per 1000 passengers), China (over 1000 km from Wuhan).

On the other hand, the picture is more homogeneous for Chinese provinces and territories over 1000 km from Wuhan (Figure 4). For such distances, air travel is practically the only option for passengers and the q indicator has a range of between 2 and 6 cases per thousand passengers. The two outliers are Tibet (with a single case in the period, but also a very low number of estimated passengers) and Heilongjiang, where the number of cases was proportionally higher already on February 3, possibly as a result of the province having entered the local transmission phase early.

**Figure 5:**
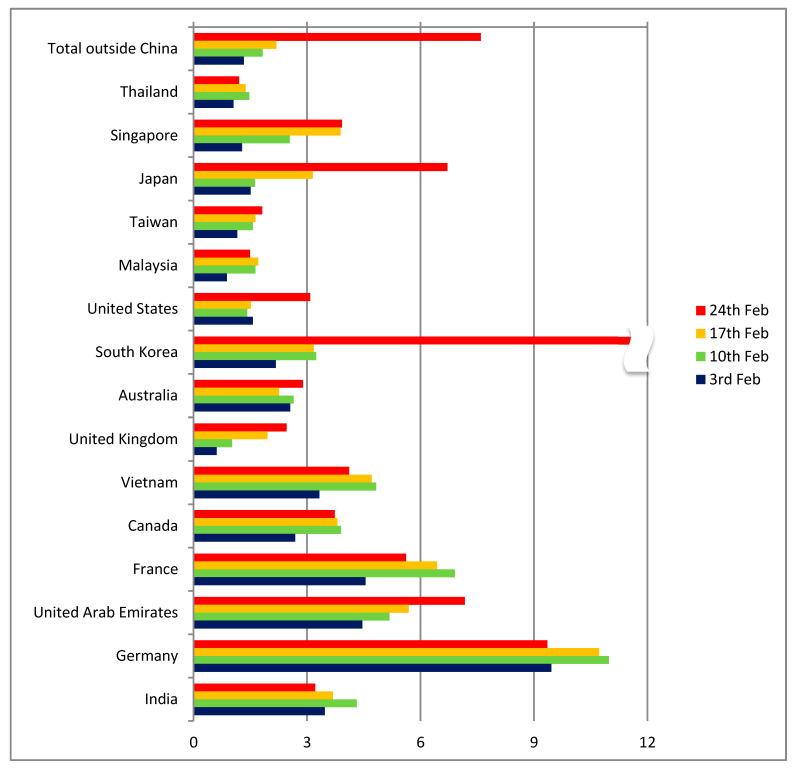
Estimated ratio of infected passengers outside China (cases per 1000 passengers).

As regards other countries apart from China (Figure 5), the indicator had a range of between 0 and 6 cases per thousand passengers until 17 February, with Germany being an outlier (but with only 16 reported cases). Up to that point, the comparison of the indicators for distant Chinese destinations (more than 1000 km from Wuhan) and countries outside China confirmed the hypothesis of diagnosed cases being proportional to the number of passengers arriving from Wuhan. Even though the absolute number of cases was very low and the ratios were unstable, the global distribution patterns seemed to follow the expected dynamics and to correspond to the air transport patterns.

Given that the majority of final destinations of passengers from Wuhan were in Mainland China, the results implied that the appearance of infected cases would follow the same pattern as that of aviation trips (Figure 1), with the risk for countries outside Asia being low. Additionally, since China had implemented an extended quarantine since the last week of January, these patterns suggested that the risk for additional cases in the rest of the world would be limited, as infections in other Chinese provinces and territories were kept under control. An additional observation that could be made at that moment was that the reporting quality in China was comparable to that of most neighboring countries, with indicator levels being at similar levels. However, surprisingly a number of countries—including Indonesia and the Philippines—report a very low number of cases compared to the expected number of passengers from Hubei. It should be noted, though, that the number of tests performed in several countries was very low and the actual number of symptomatic or asymptomatic cases may actually be much higher.

A dramatic change can be seen in the data for the 24th of February in a number of countries outside China (Figure 5). Suddenly, the number of cases in South Korea, Japan and Iran presented a pronounced spike. While South Korea and Japan were among the countries where the risk had already been identified, Iran was a case that the travel pattern analysis had completely missed. The model estimates on the number of travellers from Hubei to Iran were between 33 and 236 per month, a value too low to raise any alarm. These three countries were only the beginning of the pandemic at the worldwide level that in the course of the following 8 weeks infected more than 3 million people and caused more than 200 thousand deaths (as of 27 April 2020).

It is now known that the main reason for the rapid expansion in countries outside Asia was the appearance of local foci that were not detected initially, and the subsequent spread of the virus from them both locally and internationally. This evolution of the pandemic revealed a curious pattern: the countries that had high levels of interaction with China realized the level of risk early and applied strict control measures, resulting in a comparatively limited spread of the disease. Whereas countries that appeared to have a lower risk—since they were not in such direct contact with the area of the primary focus of the pandemic—eventually reached disease levels that exceeded those in the original focus in China (especially the USA, Spain and Italy). In that sense, our approach appears as sufficient to capture the true positives, but—as all approaches that we are aware of—may underestimate risk and lead to false negatives with a high impact. This is a general weakness of any risk assessment model that we try to explain and discuss in the following sections.

## 4. Limitations to Predictive Capacity

The capacity of aviation activity to predict the appearance of infected cases can be affected by various factors that may influence the ratio of infected passengers reaching a specific destination. In the approach presented here, we assumed—for lack of data—that the socio-economic and demographic profile of travellers to all destinations is the same. The assumed profile includes the ratio of local to visiting travellers and their age distribution, as well as their income or professional activity distribution.

Most probably, the infection rates of the local population in Hubei province are different than the one for visitors, mainly because of the difference in terms of the total time spent in the origin of the pandemic and—as a result—the period of exposure. The analysis of exportations of symptomatic cases with MERS-CoV in the period 2013–2015 [26] differentiated between locals and visitors to the Middle East, the center of the MERS outbreak. The exposure time of visitors was estimated using tourism statistics, while the exposure of locals was assumed to be equal to the upper bound of the disease incubation period (10.2 days). A model-based study of frequent flyers suggests that the probability that an asymptomatic infected person will make an international flight is higher for high-frequency travellers than for low-frequency ones [27]. One would therefore expect differences in the infection rates between destinations that serve mainly visitors to Wuhan (e.g., flights to Europe) and destinations that serve a higher proportion of locals (e.g., flights to destinations in China with employees returning to work).

The socio-economic profile of the travellers can also affect the probability of super-spreaders, individuals who due to the type or intensity of their social interactions can transmit the disease to a disproportionate number of others. A higher frequency of infections in health care settings has been observed for SARS and MERS [28]. To what concerns travel, frequent flyers have been shown to have a higher probability of being super-spreaders [1]. Visitors to Wuhan, especially ones for business purposes, probably had a higher number of social interactions at the origin than the average local inhabitant (as well as at the destination, increasing even more the probabilities of spreading the disease). A similar pattern may have been in place in the propagation from the secondary focus of Milan, where a large number of foreign visitors to commercial exhibitions or sport events may have caused the spread to other countries.

Infection during flights has been found to have a significant probability. Even though aircraft are equipped with High-Efficiency Particulate Air (HEPA) filters, which reduce the risk of transmission through the air circulation system, several studies have demonstrated that infection rates can be as high as 4.3% in a 9-h flight, and that the risk does not depend on the relative seating of contagious passengers and passengers infected in-flight [29]. Mangili and Gendreau already discussed the need for additional work on in-flight transmissions [30], but insufficient data have been made available in the meanwhile to allow a more robust conclusion.

The diagnostic capacity and protocols applied in airport controls have been discussed using the experience from SARS [31,32]. Grout et al. [33] identified several inconsistencies in infection control measures and major differences in how national and international legislation is enforced by airlines and airports. The differences in the diagnosing quality may affect the number of cases reported. However, while focusing on controls at the point of entry may improve the identification of travellers with symptoms, in the case of COVID-19 this strategy may have given a false sense of security.

Climate and local environmental conditions may also affect the speed of local transmission and can lead to deviations from the expected number of infections. Such an association has been suggested for SARS and MERS [34], but has not yet been demonstrated in the case of COVID-19. It is still not clear how the virus reacts to temperature or air pollution, especially particulates. Both aspects will probably be researched extensively in the future.

The low level of risks suggested by both aviation data and airport controls can distract attention from what—in hindsight—proved to be the main reason the pandemic reached such levels: asymptomatic cases. The number and transmission capacity of asymptomatic cases was a “known unknown” from the beginning of the outbreak. Since COVID-19 has a long incubation period (up to or even exceeding 14 days) and tests were not yet available, the number of asymptomatic carriers could not be measured or estimated even in later stages of the pandemic.

## 5. Conclusions

The frequently cited work by Colizza et al. (2007) explored the predictability of stochastic dynamic models on global disease distribution and concluded that:

“… stochastic fluctuations are less important than the constraints imposed by the transport network structure that imposes an overall pattern to the epidemic evolution. In this respect, global properties provide insights on the general features of the epidemic spreading in relation with the underlying network but do not provide adequate information on the predictability of the resulting pattern” [35].

Our empirical results from modeling the spread of COVID-19 suggest the opposite, at least as regards the first part of the statement. The stochastic fluctuations of the factors that affect the air transport-related spread of the disease introduce high levels of uncertainty and consequently limit the predictability of the resulting spatial pattern.

Aviation activity analyzed with the methodology we presented here can provide an early signal for risk. Such information is important for destinations with a high number of passengers from the origin of the pandemic, since it can correctly identify potential risks. The indicator used is also a convenient measure to compare the evolution across countries and identify changes in the direction of the trends.

Soon, however, if a large enough number of disease carriers has travelled undetected, the easiness of air travel can convert a local outburst into a global pandemic. In that case, the value of aviation-based distribution models is limited, due to their systematic underestimation of risk for destinations with low passenger volume from the origin or interactions with a secondary focus of infection.

Strict measures (quarantine, flight cancelations, travel restrictions) can reduce risks significantly but come with the cost of a large social, economic and political risk. Airport controls, especially in the case of novel diseases with unknown symptoms or high asymptomatic shares, can prove inadequate and may even give a false sense of security. International collaboration and exchange of information are important elements in managing risk. While the random effects of disease propagation cannot be controlled, monitoring the situation at the global level using reliable data can significantly improve the reaction of public authorities.

As with any risk assessment approach, the evaluation of the actual risk and the costs of the possible risk mitigation strategies can pose delicate trade-offs. However, keeping in mind that modern aviation permits connecting any two points on the planet in less than 48 h, the chances for new virulent diseases spreading around the world are high and the need for better predictive tools becomes even more urgent.

## Figures and Tables

**Table 1:** Relative shares of aviation passengers from Hubei province to major zones, January 2019 data versus estimates for January 2020.

Zone	Share in Jan. 2019 (%)	Share in Jan. 2020 (%)	Difference (Percentage Points)
China (>1000 km)	45.8	45.6	−0.2
China (700–1000 km)	41.4	40.1	−1.3
Southeast Asia	4.1	4.9	0.81
Far East Asia	3.2	3.7	0.54
China (<700 km)	3.3	3.7	0.38
Europe	0.7	0.7	0.03
Americas	0.7	0.6	−0.19
Oceania	0.4	0.4	−0.06
Rest of Asia	0.4	0.4	−0.01
Africa	0.1	0.1	0

**Table 2:** Number of reported cases, China (<700 km from Wuhan).

Province	3rd February 2020	10th February	17th February	24th February
Hubei	11,177	31,728	59,989	64,287
Henan	566	1105	1257	1271
Hunan	521	912	1007	1016
Anhui	408	860	982	989
Jiangxi	391	804	933	934
Hebei	113	239	302	311
**Total**	**13,176**	**35,648**	**64,470**	**68,808**

**Table 3:** Estimated passengers and number of reported cases, China (700–1000 km from Wuhan).

Province	Estimated Aviation Passengers Jan. 2020	3rd Feb. 2020	10th Feb.	17th Feb.	24th Feb.
Guangdong	109,812	725	1177	1328	1345
Zhejiang	56,054	724	1117	1172	1205
Chongqing	35,278	312	489	553	575
Jiangsu	11,376	271	515	629	631
Shandong	49,634	259	487	543	755
Shanghai	56,127	203	303	333	335
Fujian	71,460	179	267	292	293
Shaanxi	24,427	66	219	240	245
Shanxi	26,472	66	122	130	132
**Total**	**440,641**	**2805**	**4696**	**5220**	**5516**

**Table 4:** Estimated passengers and number of reported cases, China (over 1000 km from Wuhan).

Province	Estimated Aviation Passengers Jan. 2020	3rd Feb. 2020	10th Feb.	17th Feb.	24th Feb.
Sichuan	60,813	255	417	508	527
Beijing	57,573	191	342	387	399
Guangxi Zhuang	38,060	127	215	242	251
Heilongjiang	14,709	118	360	464	480
Yunnan	60,576	114	153	172	174
Liaoning	51,240	73	111	121	121
Hainan	87,056	71	144	163	168
Tianjin	26,128	56	105	128	135
Gansu	19,432	51	86	91	91
Guizhou	28,850	46	127	146	146
Inner Mongolia	13,487	34	58	73	75
Jilin	11,793	31	81	89	93
Ningxia Hui	11,969	31	53	70	71
Xinjiang Uygur	17,858	24	55	76	76
Hong Kong	7597	15	36	61	79
Qinghai	6581	13	18	18	18
Macau	6098	8	10	10	10
Tibet	3225	1	1	1	1
**Total**	**523,046**	**1259**	**2372**	**2820**	**2915**

**Table 5:** Estimated passengers and number of reported cases, other main countries.

Country	Estimated Aviation Passengers Jan. 2020	3rd Feb. 2020	10th Feb.	17th Feb.	24th Feb.
Japan	12,094	20	26	59	144
Thailand	16,428	19	32	35	35
Singapore	12,797	18	43	77	89
South Korea	6297	15	27	31	763
Australia	4286	12	15	15	22
Taiwan	8688	11	18	22	28
United States	6397	11	12	15	35
Germany	964	10	14	16	16
Malaysia	8322	8	18	22	22
Vietnam	2194	8	14	16	16
France	1204	6	11	12	12
United Arab Emirates	1022	5	7	9	13
Canada	1359	4	7	8	9
United Kingdom	2979	2	4	9	13
Philippines	2107	2	3	3	3
India	526	2	3	3	3
Italy	760	2	3	3	132
Russia	470	2	2	2	2
Cambodia	5775	1	1	1	1
Indonesia	6644	0	0	0	0
Pakistan	733	0	0	0	0
New Zealand	763	0	0	0	0
Nepal	309	0	1	1	1
Sri Lanka	153	0	1	1	1
Rest of the World	5376	0	0	8	104
**Total outside China**	**108,647**	**158**	**262**	**368**	**1464**

**Figure A1:**
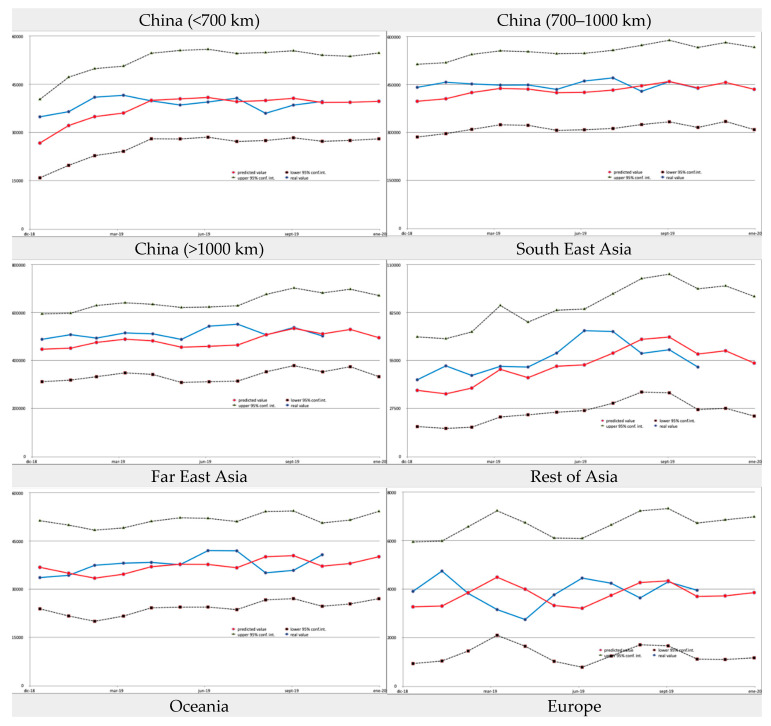

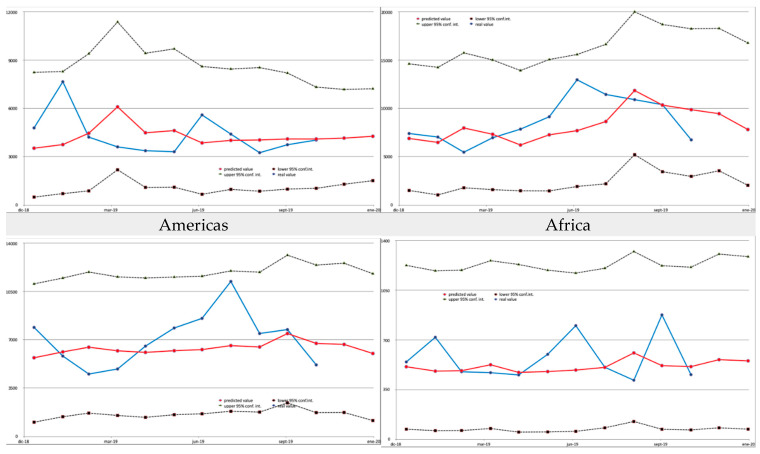
Predicted and real numbers of monthly aviation passenger per region.
